# Genome-wide characterization and expression analysis suggested diverse functions of the *mechanosensitive channel of small conductance-like* (*MSL*) genes in cereal crops

**DOI:** 10.1038/s41598-020-73627-7

**Published:** 2020-10-06

**Authors:** Amandeep Kaur, Mehak Taneja, Shivi Tyagi, Alok Sharma, Kashmir Singh, Santosh Kumar Upadhyay

**Affiliations:** 1 Department of Botany, Panjab University, Chandigarh, 160014 India; 2 Department of Biotechnology, Panjab University, Chandigarh, 160014 India

**Keywords:** Computational biology and bioinformatics, Plant sciences, Functional genomics, Genomics, Plant biotechnology

## Abstract

Mechanosensitive ion channels are pore-forming transmembrane proteins that allow ions to move down their electrochemical gradient in response to mechanical stimuli. They participate in many plant developmental processes including the maintenance of plastid shape, pollen tube growth, etc. Herein, a total of 11, 10, 6, 30, 9, and 8 *MSL* genes were identified in *Aegilops tauschii, Hordeum vulgare, Sorghum bicolor, Triticum aestivum, Triticum urartu,* and *Zea mays,* respectively*.* These genes were located on various chromosomes of their respective cereal, while *MSL*s of *T. urartu* were found on scaffolds. The phylogenetic analysis, subcellular localization, and sequence homology suggested clustering of MSLs into two classes. These genes consisted of *cis*-regulatory elements related to growth and development, responsive to light, hormone, and stress. Differential expression of various *MSL* genes in tissue developmental stages and stress conditions revealed their precise role in development and stress responses. Altered expression during CaCl_2_ stress suggested their role in Ca^2+^ homeostasis and signaling. The co-expression analysis suggested their interactions with other genes involved in growth, defense responses etc. A comparative expression profiling of paralogous genes revealed either retention of function or pseudo-functionalization. The present study unfolded various characteristics of MSLs in cereals, which will facilitate their in-depth functional characterization in future studies.

## Introduction

All organisms including bacteria, plants and animals, receive mechanical signals from both internal (osmotic pressure and plasma membrane distortion) and external environment (gravity, touch, sound)^1^. They respond to these signals by activating various channel proteins. A special type of channels that open and close in response to mechanical forces are known as mechanosensitive (MS) ion channels or stretch-gated ion channels^1^. They are pore-forming transmembrane (TM) proteins, which allow the movement of ions across the plasma membrane down to their electrochemical gradient. However, in plants, MS channels also act in response to signals related to developmental processes, such as cell wall damage, pollen tube growth, plant–pathogen interactions, and lateral root emergence^2–4^. Initially, MS channels were recognized during the electrophysiological characterization of the *Escherichia coli* cell envelope^5–7^. Based on the ionic conductance, the MS channels were divided into two main groups: mechanosensitive channels of large conductance (MscL) and mechanosensitive channels of small conductance (MscS)^8,9^. The ionic conductance of MscL and MscS corresponds to about three and one nanosiemens, respectively^1^. Previous studies suggested that MscL and MscS have no ion specificity while showing permeability to very small charged molecules such as potassium glutamate, proline^10^, etc. During hypo-osmotic shock, the bacterial MS channels act as a safety valve by allowing the diffusion of ions from the cell and prevent its bursting^5,11,12^.

Both MscL and MscS homologs are distributed in archaea, bacteria, and fungi, while MscS channels homologs are also found in algae and plants. A maximum of 10 MscS-Like (MSL) channels are found in model plant *Arabidopsis thaliana,* which are classified into two main classes: Class I MSL and Class II MSL^13,14^. Class I MSLs are found to be localized either in mitochondrial membrane or plastid envelop and show sequence similarity with MscS channels of bacteria. However, Class II MSLs are predicted to be present in the plasma membrane. Further, the characterization studies of MSL channels of *A. thaliana* suggested that AtMSL9 and AtMSL10 perform mechanosensitive activities in the plasma membrane of root cells^15^. Upon exposure to various stimuli, the AtMSL9 and AtMSL10 may increase cytosolic Ca^2+^ concentration in a small amount or they may depolarize the membrane, leading to activation of other Ca^2+^ channels^16^. In addition to it, AtMSL2 and AtMSL3 play a significant role in the regulation of plastid size and Z- rings formation^17,18^. Besides, AtMSL8 promotes pollen grain hydration and germination^19^. The *MSL* genes have been reported in other crops such as *Oryza sativa, Phaseolus vulgaris, Cicer arietinum*, etc.^20–22^. Due to their crucial role in plants during stress conditions and developmental stages, it is important to explore this gene family in other crop plants. In the current study, we identified and characterized *MSL* genes in six cereals such as *Aegilops tauschii, Hordeum vulgare, Sorghum bicolor, Triticum aestivum, Triticum urartu* and *Zea mays*. The identified genes and proteins were analyzed for structural configurations, chromosomal and subcellular distribution, *cis*-regulatory elements, duplication events, and phylogenetic relatedness. The expression profiling of *MSL* genes was carried out in abiotic and biotic stress conditions and, various tissue developmental stages using high throughput RNA sequence data. Further, we performed qRT-PCR for the expression study of a representative *TaMSL* gene from each phylogenetic group under CaCl_2_ stress conditions. Co-expression studies of *MSL* genes provided information about their interacting partners and their contribution to various biological processes of plants. This study provided valuable information about *MSL* genes, which will be useful in the future for their functional characterization.

## Results and discussion

### Identification and chromosomal distribution

A total of 74 putative *MSL* genes were identified after an extensive search, out of which diploid crops including *Ae. tauschii, H. vulgare* , *S. bicolor, T. urartu*, and *Z. mays* consisted of 11, 10, 6, 9, and 8 genes respectively (Supplementary File S1). *S. bicolor* have less number of genes as compared to other studied crops, which might be due to its small genome size^23^. We identified a maximum of 30 *MSL* genes in *T. aestivum* (Supplementary File S1), which could be due to its allohexaploid nature^24^. The previous findings have suggested 10 *MSL* genes in *A. thaliana*^13,14^, six in each *O. sativa*^20^ and *C. arietinum*^22^, and nine in *P. vulgaris*^21^. Therefore, the number of genes identified in the present study in six cereal plant species showed consistency with previous studies.

**Figure 1:**
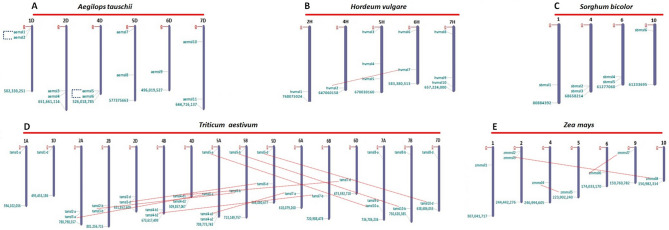
Chromosomal localization and duplication analysis of *MSL* genes in five cereals. The *AeMSL* genes are distributed on 6 chromosomes of *Ae. tauschii* (**A**), *HvMSL* genes on 5 chromosomes of *H. vulgare* (**B**), *SbMSL* genes on 4 chromosomes of *S. bicolor* (**C**), *TaMSL* genes on 16 chromosomes of A, B and D subgenomes of *T. aestivum* (**D**), and *ZmMSL* genes on 7 chromosomes of *Z. mays* (**E**). Blue and Red dotted lines point toward the existence of tandemly and segmentally duplicated genes, respectively.

The chromosomal map of *MSL* genes indicates their variable distribution on six, five, four, and seven chromosomes of *Ae. tauschii, H. vulgare, S. bicolor*, and *Z. mays,* respectively (Fig. 1A–C,E). The *MSL* genes of *T. aestivum* were distributed on various chromosomes and subgenomes (A, B, and D) except chromosome 1B, 3A, 3B, 3D, and 4A. The highest number of *TaMSL* genes were found on chromosome 5A (Fig. 1D). However, in the case of *T. urartu*, scaffold locations of *MSL* genes were predicted. The distribution of *MSL* genes on various chromosomes has also been reported in other crops such as *O. sativa, P. vulgaris*, *C. arietinum*^20–22^.

### Prediction of homeologs and duplication events

According to previous studies, each progenitor subgenome (i.e. A, B, and D) has contributed to the formation of a large number of gene families in *T. aestivum*^25–27^ . We have identified 10 clusters of homeologous *TaMSL* genes and found that most of these clusters consisted of at least one gene from each subgenome. However, the homeologous group *TaMSL1* was found on only A and D subgenome, while *TaMSL9* was located on only A subgenome. The number of homeologous groups of *TaMSL* genes was found comparable to the number of *MSL* genes identified in the diploid plant species such as *A. thaliana*, *P. vulgaris*, and *C. arietinum*^13,14,21,22^_._ .

The duplication events lead to the origin of paralogous genes during evolution and play a major role in the expansion of gene families. They are essential for genetic variability and in gaining additional roles of genes, which further promote adaptation and speciation^28–30^. We analyzed duplication events (DEs) to study their contribution in the evolution of the *MSL* gene family. A total of 16 DEs were identified, out of which two were tandem duplication events (TDEs) and 13 were segmental duplication events (SDEs) (Supplementary File S2). One duplication event (*TuMSL4-TuMSL7*) found in *T. urartu* could not be categorized as TDE or SDE due to scaffold location of these genes (Supplementary File S2). Two TDEs (*AeMSL1-AeMSL2; AeMSL5-AeMSL6*) were found in *Ae. tauschii,* while one (*HvMSL2-HvMSL7*), nine (*TaMSL3-A-TaMSL6-A; TaMSL3-B-TaMSL6-B; TaMSL3-D-TaMSL6-D*, etc.) and three (*ZmMSL3-ZmMSL8; ZmMSL4-ZmMSL5; ZmMSL6-ZmMSL7*) SDEs were found in *H. vulgare, T. aestivum* and *Z. mays,* respectively (Fig. 1A,B,D,E)*.* However, no duplication event was found to occur in the genome of *S. bicolor*. The DEs in *MSL* genes were earlier reported in some plants such as *O. sativa, P. vulgaris* and *C. arietinum*^20–22^. The variation in number of duplication events might be due to different genome size and ploidy level. For instance, the complex genome of *T. aestivum* is responsible for the occurrence of a large number of duplication events*.* The numerous incidences of SDEs have been attributed to the enlargement of gene families in *T. aestivum* that also supported the results obtained in the present study^31–33^.

### Ka/Ks analysis

We have calculated the values of Ka, Ks and Ka/Ks ratio to know about the evolutionary divergence between the paralogous *MSL* genes (Table 1). The Ka/Ks ratio of all the duplicated gene pairs was less than one, which suggested negative or purifying selection pressure throughout the evolution. Due to non-availability of complete sequence of *AeMSL2*, it was not possible to perform Ka/Ks analysis of duplicated gene pair *AeMSL1*-*AeMSL2*.

**Table 1:** The Ka/Ks ratio and divergence time of duplicated *MSL* gene pairs.

Gene A	Gene B	Ka	Ks	Ka/Ks	T = Ks/2r	Selection pressure
*AeMSL6*	*AeMSL5*	0.0625	0.2396	0.2608	18.4	Purifying
*HvMSL2*	*HvMSL7*	0.0930	0.4718	0.1972	36.3	Purifying
*TaMSL3-A*	*TaMSL6-A*	0.1433	1.3404	0.1069	103.1	Purifying
*TaMSL3-B*	*TaMSL6-B*	0.1417	1.4309	0.0990	110.1	Purifying
*TaMSL3-D*	*TaMSL6-D*	0.1380	1.3358	0.1033	102.8	Purifying
*TaMSL4-A1*	*TaMSL7-A*	0.0786	0.4318	0.1821	33.2	Purifying
*TaMSL4-B2*	*TaMSL7-B*	0.0986	0.4010	0.2458	30.8	Purifying
*TaMSL4-D1*	*TaMSL7-D*	0.0982	0.4136	0.2375	31.8	Purifying
*TaMSL5-A*	*TaMSL10-A*	0.1940	0.5637	0.3442	43.4	Purifying
*TaMSL5-B*	*TaMSL10-B*	0.1982	0.5867	0.3377	45.1	Purifying
*TaMSL5-D*	*TaMSL10-D*	0.2081	0.5810	0.3581	44.6	Purifying
*TuMSL4*	*TuMSL7*	0.0816	0.4657	0.1751	35.8	Purifying
*ZmMSL3*	*ZmMSL8*	0.0287	0.3195	0.0898	24.6	Purifying
*ZmMSL4*	*ZmMSL5*	0.0505	0.2499	0.2022	19.2	Purifying
*ZmMSL6*	*ZmMSL7*	0.0421	0.2184	0.1927	16.8	Purifying

*Ka* non-synonymous substitutions per non-synonymous site, *Ks* synonymous substitutions per synonymous site, *T* divergence time.

Further, the divergence time of DEs was also calculated and it was predicted to be in the range of 16 to 110 Million years ago (MYA). The maximum and minimum divergent time of DEs were found in *T. aestivum* and *Z. mays*, respectively (Table 1). In previous study, it was estimated that main DEs were occurred ~ 67 MYA and ~ 11 MYA in *H. vulgare* and *Zea mays*, respectively^34^. The predicted duplications in these plants were in similar range with slight deviation, as it was ~ 36MYA in *HvMSLs*, and ~ 16–24 MYA in *ZmMSLs* (Table 1). In case of *TaMSL* paralogs, the DEs were predicted in the diverse range from ~ 30 to 110 MYA. The results suggested the occurrence of these DEs before the hybridization of A, B and D subgenomes that occurred in the range of ~ 0.5–6 MYA^24^. Moreover, higher rate of sequence divergence among the duplicated gene pairs during evolution could also be the region for the deviation in the years of predicted duplication events, as it was completely based upon sequence analysis.

### Phylogenetic analysis

**Figure 2:**
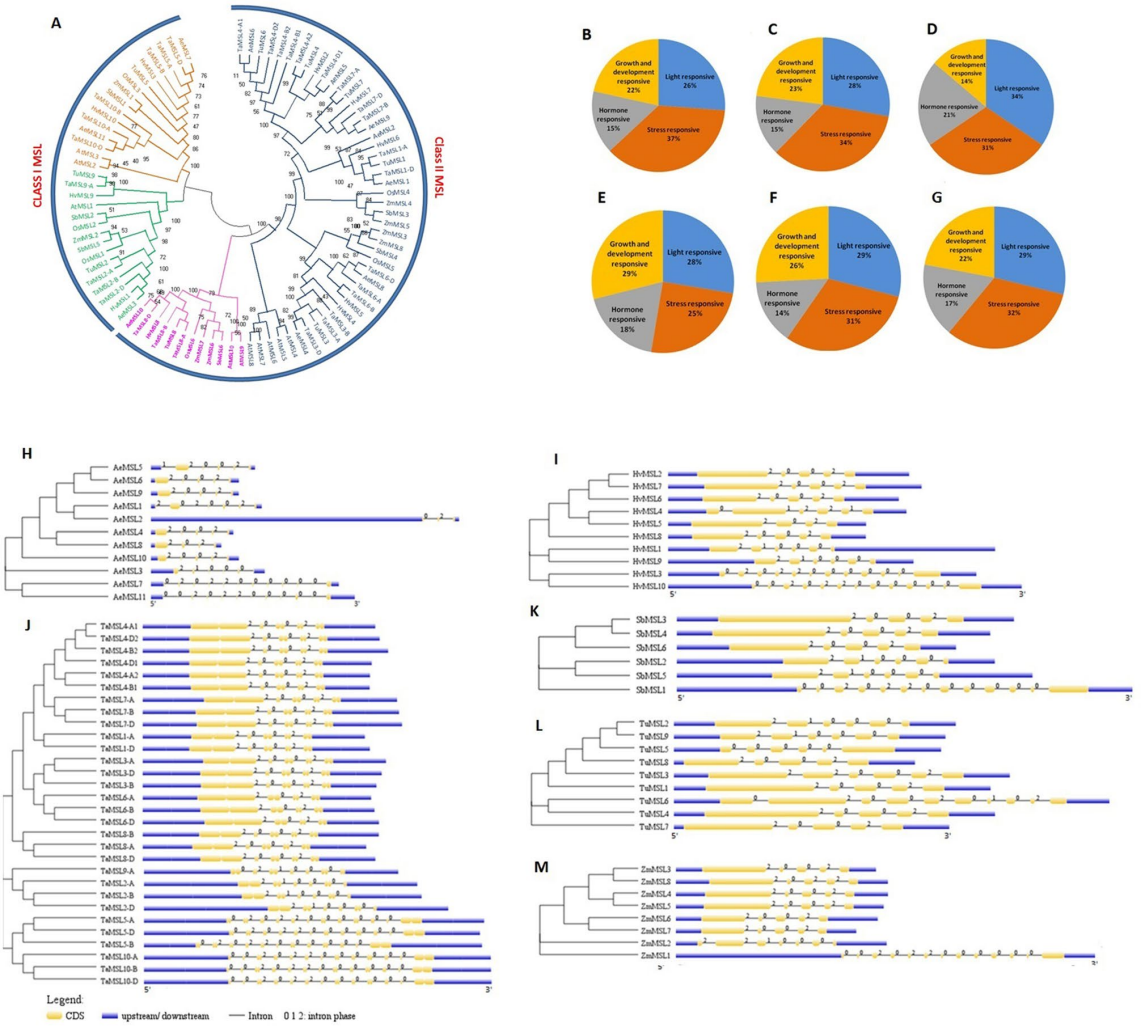
Phylogenetic tree, *cis*-regulatory element analysis, and gene structure (exon–intron organization and intron phases) analysis. (**A**) Shows a phylogenetic tree constructed by the neighborhood joining method with 1000 bootstraps using MEGA7.0 software. The tree shows two major classes i.e. class I and class II, which further divided into sub-classes highlighted with different colors. (**B–G**) Show the percentage of *Cis*-regulatory elements including light-responsive elements, stress-responsive elements, hormone-responsive elements, and growth and development responsive elements of MSL genes of *Ae. tauschii* (**B**), *H. vulgare* (**C**), *S. bicolor* (**D**), *T. aestivum* (**E**), *T. urartu* (**F**), and *Z. mays* (**G**). (**H**–**M**) Show the structure of MSL genes of *A. tauschii* (**H**), *H. vulgare* (**I**), *T. aestivum* (**J**)*, S. bicolor* (**K**)*, T. urartu* (**L**)*, and Z. mays* (**M**) prepared by using GSDS 2.0. server.

The phylogenetic tree was prepared to study the evolutionary relatedness among the MSL proteins of *Ae. tauschii, A. thaliana*, *H. vulgare, O. sativa, S. bicolor, T. aestivum, T. urartu*, and *Z. mays* using the full length protein sequences. In the phylogenetic tree, MSL proteins were clustered into two main classes i.e. class I and II (Fig. 2A). Similar classification of proteins has also been reported in *A. thaliana*^17^ *O. sativa*^20^, *P. vulgaris*^21^ and *C. arietinum*^22^. The class I and class II consisted of 31 and 59 MSL proteins, respectively. Two MSL proteins of *Z. mays*, three of each *Ae. tauschii, A. thaliana, O. sativa, S. bicolor* and *T. urartu*, four of *H. vulgare*, and 10 of *T. aestivum* were clustered in class I. While, three MSL proteins of each *O. sativa* and *S. bicolor*, six of each *H. vulgare, T.urartu* and *Z. mays*, seven of *A. thaliana*, eight of *Ae. tauschii,* and 20 of *T. aestivum* showed close relationships and found in class II of phylogenetic tree (Fig. 2A).

The majority of paralogous genes were found close to each other, for instance, *ZmMSL3-ZmMSL8*, *ZmMSL4-ZmMSL5*, *AeMSL1-AeMSL2*, *TaMSL3-A-TaMSL6-A*, etc. The structural relatedness among the paralogous genes might be responsible for close clustering. The homeologous *TaMSL* of *T. aestivum* were tightly clustered due to their sequence resemblance at high bootstrap value (Fig. 2A). This was also in confirmation with the used nomenclature of *TaMSL* genes.

### Promoter analysis

Promoter analysis suggested the occurrence of a large number of *cis*-regulatory elements in the promoter regions of *MSL* genes, which were responsive to light, hormone, stress, and growth and development (Fig. 2B–G). Some light-responsive elements including Sp1, G-box, G-Box, ACE, and GT1-motif were identified in most of *MSL* genes. STRE, as-1, MYB, MYC, WRE3, GC-motif, DRE core, TC-rich repeats, MYB recognition site, etc. were commonly occurring stress-responsive elements in *MSL* genes. Hormone-responsive elements such as ABRE, CGTCA-motif, TGACG-motif, ABRE3a, and ABRE4 were most frequently found in *MSL* genes. Furthermore, the growth and development related *cis*-regulatory elements including; CAT-box, Myb-binding site, CCGTCC-box, CCGTCC motif, AAGAA-motif was detected in most of the *MSL* genes. In *Ae. tauschii,* 37% *cis*-elements were found to be responsive to stress, 26% to light, 22% to growth and development, and 15% to hormones (Fig. 2B). In *H. vulgare*, 34% *cis*-elements were responsive to stress, 28% to light and 23% to growth and development, and 15% to hormone (Fig. 2C). In *S. bicolor,* 34% of *cis*-elements were found as light-responsive, 31% as stress-responsive, 21% as hormone-responsive, and 14% as growth and development responsive (Fig. 2D). In *T. aestivum*, 29% *cis*-elements were predicted to be involved in growth and development, and 28% responsive to light, 25% responsive to stress, 18% to hormone (Fig. 2E). In *T. urartu,* 31% elements were responsive to stress, 29% to light, 26% to growth and development, and 14% hormone-responsive (Fig. 2F). Similarly, in the case of *Z. mays*, we found that 32% *cis*-elements were responsive to stress, 29% to light, 22% to growth and development, and 17% to hormone (Fig. 2G). Besides, we found that the arrangement of *cis*-regulatory elements varies in each *MSL* gene. Enrichment of *cis*-regulatory elements in the *MSL* gene promoters revealed their numerous functions in each cereal.

### Gene structure analysis

The exon/intron organization and intron phases were examined to get information about the structural features of *MSL* genes. The number of exons ranged from 3 to 12 in *Ae. tauschii,* 4 to 13 in *H. vulgare* and *T .aestivum*, 5 to 13 in *S. bicolor* and *Z. mays,* and 5 to 10 in *T. urartu*. (Supplementary File S3, Fig. 2H–M). The majority of *MSL* genes carried five exons for each cereal except *T. urartu,* where six exons were present in the majority of genes. Further, we found that the class I *MSL* genes consisted of 6–13 exons, while class II *MSL* genes consisted of 3 to 10 exons in all the studied crops. The maximum number of introns were present in 0 phase followed by 2 phase and then 1 phase, which indicated the conserved architecture of *MSL* genes (Fig. 2H–M). The previous studies regarding gene structure of *MSLs* suggested the number of exons from 5 to 19 in *O. sativa*^20^, 4 to 12 in *P. vulgaris*^21^ and 5 to 8 in *C. arietinum*^22^ which showed consistency with the structural organization of *MSLs* in our studied crops.

### Protein characterization

The physicochemical properties of MSL proteins were analyzed using computational approaches. The predicted average length, molecular weight (MW) and isoelectric point (pI) of MSL proteins from *Ae. tauschii, H. vulgare, S. bicolor, T. aestivum, T. urartu* and *Z. mays* ranged from 680 to 782 AA, 73.4 to 90.6 kDa and, 9.1 to 9.7, respectively (Supplementary File S3). These findings were similar to the previously reported MSL proteins in other plants^20–22^.

In each cereal crop, class I MSL proteins consisted of five or six TM helices, while class II MSL proteins carried five to eight TM helices (Supplementary File S3). However, AeMSL2 and TuMSL5 protein exhibited the occurrence of only one and two TM helices, respectively, which could be due to the partial sequence of these genes. Previous studies have suggested the presence of five TMs in class I and six TMs in class II MSLs of *A. thaliana*^14^ and *O. sativa*^22^, which were comparable to our findings.

The subcellular localization prediction of MSL proteins of each cereal suggested that class I MSL proteins were either located on chloroplastic envelop or mitochondrial membrane, while class II proteins were located on the plasma membrane (Supplementary File S3). Similar results have been reported in other crops including *A. thaliana*, *O. sativa* , *P. vulgaris* and *C. arietinum*^14,20–22^.

**Figure 3:**
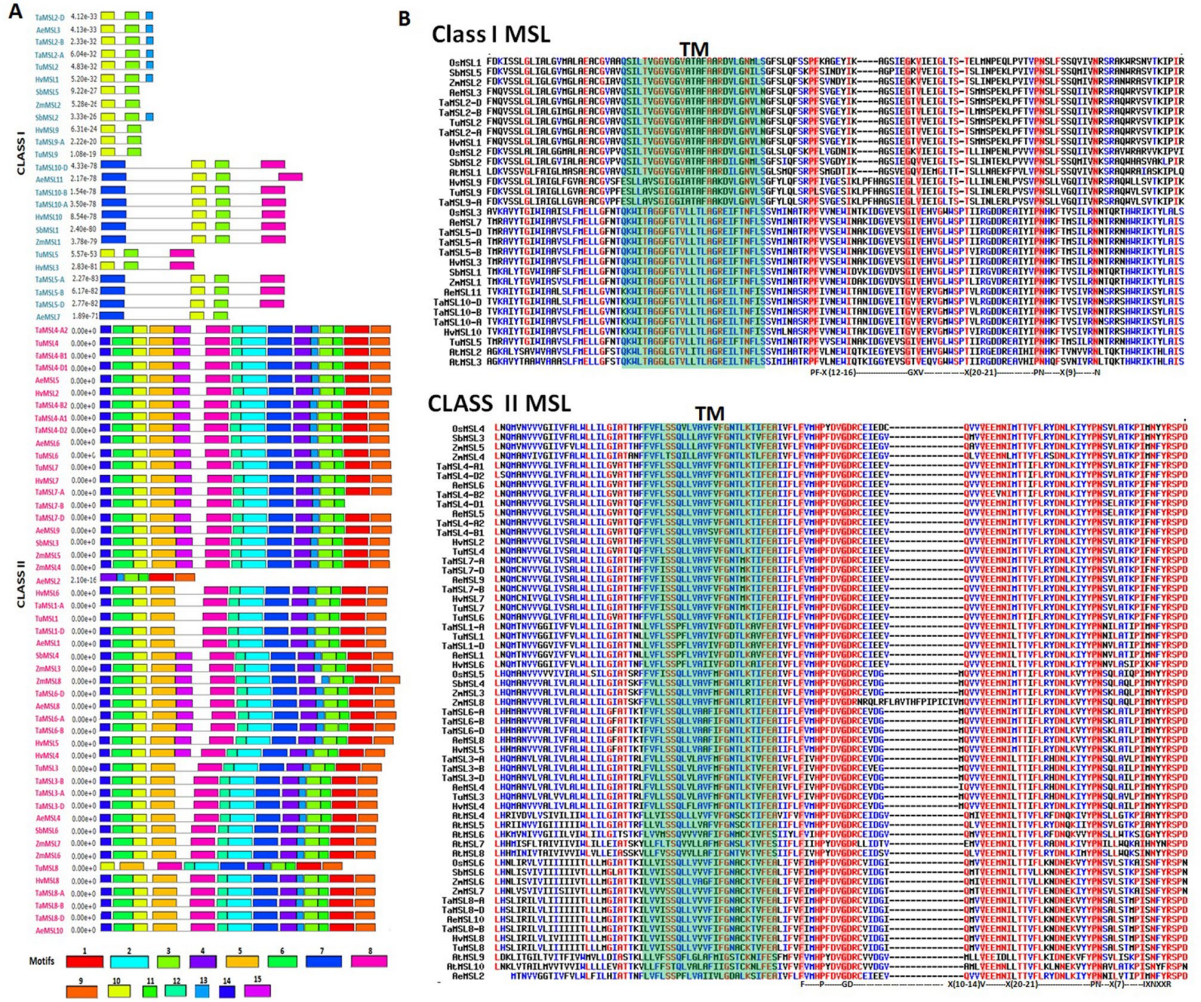
Motif analysis and multiple sequence alignment of MSL proteins. (**A**) Shows the distribution of 15 conserved motifs in MSL proteins. The colored boxes represent these conserved motifs constructed by the MEME suite. (**B**) Shows multiple sequence alignments with the conserved transmembrane region (TM) and a conserved motif in each class of MSL proteins.

The functionality of a protein depends upon the characteristic features of its domain. Pfam analysis suggested that the MS-channel domain (PF00924) was present in all MSL proteins of *Ae. tauschii, H. vulgare, S. bicolor, T. aestivum, T. urartu* and *Z. mays* (Supplementary File S4)*.* Further, the occurrence of 15 conserved motifs was examined among MSL proteins of each cereal. Motif 3 present in MS channel domain was found to be highly conserved, due to its occurrence in all the MSL proteins. Motif 10 was also distributed in all proteins except AeMSL2. The majority of identified motifs were present in class II MSL proteins, while motifs 3, 7, 8, 10, and 13 were present in the members of class I MSL (Fig. 3A). Furthermore, protein kinase C phosphorylation site, casein kinase 2 phosphorylation site, tyrosine kinase site could be detected in the motifs 1, 2, 4, 5,7, 8, and 9. These motifs were predominately found in most of class II proteins and few of class I proteins. These finding suggested that these proteins might also be responsible for phosphorylation. Similar results have been reported in other crops such as *O. sativa*^20^*, P. vulgaris*^21^ and *C. arietinum*^22^*.*

The identified MSL proteins in our present study were aligned along with MSL protein sequences of *A. thaliana* and *O. sativa,* to study the conserved residues among them (Fig. 3B). A conserved TM region was found in the C-terminal half of the MSL proteins of both the classes. Further, motifs PF(X12–16)GXV(X20–21)PN(X9)N and F(X3)P(X3)GD(X10–14)V(X20–21)PN(X7)IXNXXR were also found conserved at C-terminus in class I and class II MSL proteins, respectively (Fig. 3B). The C-terminal conserved TM domain of class I MSLs exhibited similarity with TM3 of *E. coli* MscS and similar findings has also been described in other crops^13,20–22^. Moreover, the TM domains of class I MSL proteins consisted of high proportion of glycine and alanine residues, while the class II MSL proteins had large hydrophobic side chains (Fig. 3B). Similar conserved domain and motifs were reported in MSL proteins of *A. thaliana*, *O. sativa*, *P. vulgaris*, and *C. arietinum*^14,20–22^.

### Expression analysis in tissue development stages

**Figure 4:**
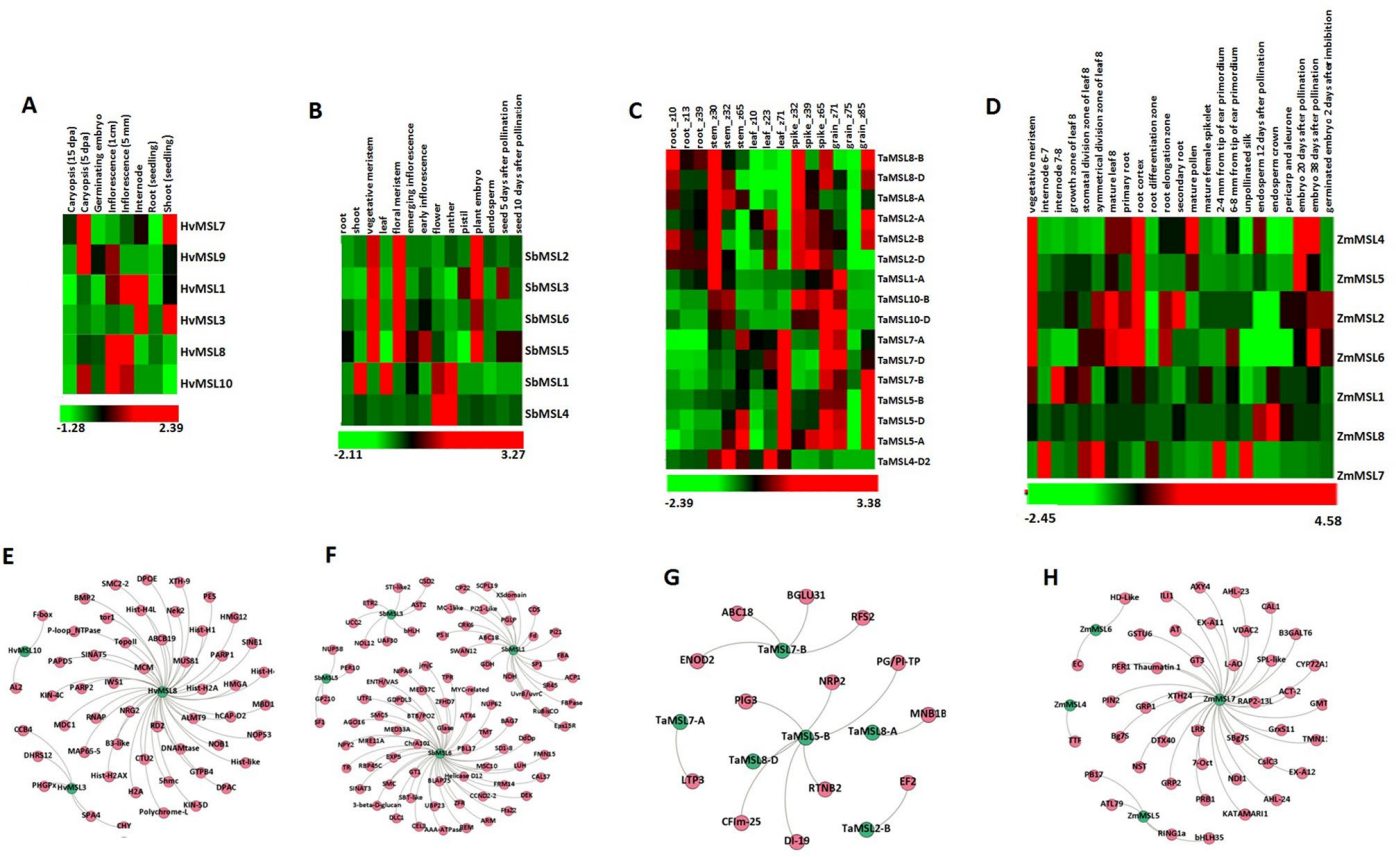
Expression profiling in various tissue developmental stages and co-expression analysis of *MSL* genes of *H. vulgare, S. bicolor, T. aestivum*, and *Z. mays*. Heat maps (**A**–**D**) show the expression profiling of *MSL* genes of *H.vulgare* (**A**), *S. bicolor* (**B**), *T. aestivum* (**C**) and, *Z. mays* (**D**). (**E–H**) Show the gene interaction networks of *MSL* genes of *H. vulgare* (**E**), *S. bicolor* (**F**), *T. aestivum,* and (**G**) *Z. mays* (**H**), based on co-expression analyses and generated using Gephi 0.9.1.

Expression analysis of *MSL* genes was carried out to study their involvement in the developmental processes of plants. We performed expression analysis in *H. vulgare, S. bicolor, T. aestivum* and *Z. mays* (Supplementary File S5, Fig. 4A–D). We selected those *MSL* genes for analysis, which showed expression ≥ 2 FPKM in a minimum of one developmental stage  (Supplementary File S5). In *H. vulgare,* most of the *HvMSLs* exhibited expression in various development stages. *HvMSL1*, *HvMSL8, HvMSL9* and *HvMSL10* showed prominent expression in reproductive tissues including inflorescence, caryopsis, germinating embryo that suggested its involvement in the development of reproductive tissues (Supplementary File S5, Fig. 4A). However, higher expression of *HvMSL3* in shoot, root and internode indicated their role in vegetative tissue development.

In *S. bicolor*, *SbMSL1* exhibited significant expression in shoot, leaf, flower, anther, and emerging inflorescence, which revealed its involvement in the development of both vegetative and reproductive tissues. Moreover, *SbMSL3, SbMSL5*, and *SbMSL6* showed expression in most of the tissues or organs. *SbMSL3* and *SbML6* exhibited elevated expression in the vegetative and floral meristem, while *SbMSL3* also exhibited enhanced expression in plant embryo and seed 5 day after pollination (DPA). *SbMSL2* showed less expression in almost all the tissues. *SbMSL4* was found to be expressed only in anther and flower suggested their participation in the development of reproductive organs (Supplementary File S5, Fig. 4B).

In *T. aestivum*, 16 *TaMSL* genes showed expression in various tissue developmental stages (Supplementry File S5, Fig. 4C). The majority of *TaMSL* genes were highly expressed in spike followed by the stem, leaf, grain, and root tissues. Moreover, these *TaMSL* genes exhibited higher expression in the later stages of tissue development. On the other hand, it was found that *TaMSL5-B* and *TaMSL5-D* showed higher expression in all the tissue development stages as compared to the rest of *TaMSL* genes. *TaMSL2* and *TaMSL8* group genes exhibited higher expression in all the developmental stages of root tissue, which suggested that they might be preferable involved in absorptive transportation of related ions in root.

In case of Z. mays, *ZmMSL1, ZmMSL2, ZmMSL5* and *ZmMSL8* showed significant expression in all the tissues development stages, which suggested their role throughout the plant development. Moreover, *ZmMSL5* exhibited quite higher expression in some tissues like tip of ear primordium, embryo 20 DPA, etc. and *ZmMSL7* was highly expressed in various reproductive tissues (Supplementary File S5, Fig. 4D).

The significant expression of the majority of *MSL* genes in various tissue developmental stages suggested their roles in the growth and development. In the case of both *A. thaliana* and *O. sativa, *the majority of *MSL *genes were expressing in various tissue/organs throughout the plant life^14,35^. *OsMSL3 *specifically shows a characteristically high expression in almost all the tissues and organs of *O. sativa*^20^. In the present study, the orthologous *TaMSL5 *group genes were also high expressing in one or more stages of the tissue development in leaf, stem, spike, and grain. Furthermore, in the case of *H. vulgare*, *S. bicolor, Z. mays*, the orthologous genes *HvMSL3, SbMSL1, and ZmMSL1* were also high expressing in all the developmental stages (Fig. 4A–D). Moreover, the majority of the *OsMSL* genes of rice are significantly expressing in reproductive stages^20^ . In the present study, several *MSLs* including *HvMSL8, SbMSL1, SbMSL4, SbMSL6, ZmMSL7* and *TaMSL5* and *TaMSL7* group genes were highly expressed in reproductive stages. The putative role of *MSL* genes in plant development was further reinforced through the occurrence of various growth and development responsive *cis*-regulatory elements like CCGTCC-box, CCGTCC motif, CTAG-motif, Circadian, CAT-box, etc.

### Co-expression analysis

We analyzed the co-expression of *MSL* genes with other genes of their respective crop in tissue development stages of *H. vulgare*, *S. bicolor*, *T. aestivum* and *Z. mays* (Supplementary File S6, Fig. 4E–H). In *H. vulgare*, three *HvMSL* genes including *HvMSL3, HvMSL10,* and *HvMSL8* were found to be co-expressed with 65 transcripts. *HvMSL8* showed co-expression with the maximum number of transcripts (56) which encoded Histone H4, a serine-threonine kinase, DNA topoisomerase 2, E3 ubiquitin- ligase, etc. (Supplementary File S6, Fig. 4E).

In the case of *S. bicolor*, four *SbMSL* genes revealed their co-expression with a total of 96 transcripts encoding ethylene receptor 2, Zinc-finger homeodomain, subtilisin-like protease, callose synthase 7, etc. (Supplementary File S6, Fig. 4F). In *T. aestivum*, six *TaMSL* genes showed co-expression with 21 transcripts encoding early nodulin 2, elongation factor 2, dehydration-induced 19, Beta-glucosidase 31, etc. (Supplementary File S6, Fig. 4G). Similarly, in *Z. mays*, four *ZmMSL* genes were co-expressing with 53 transcripts, among them *ZmMSL7* exhibited co-expression with 36 transcripts. These transcripts encoded LRR receptor-like serine-threonine kinase, L-ascorbate oxidase, E3 ubiquitin-ligase, Peroxidase 1, etc. (Supplementary File S6, Fig. 4H). The gene ontology (GO) mapping of co-expressed transcripts of *H. vulgare*, *S. bicolor*, *T. aestivum* and *Z. mays,* suggested their involvement in oxidative stress (GO: 0006979), oxidation–reduction process (GO: 0055114), iron ion binding (GO: 0005506), Zinc ion binding (GO: 0008270), electron carrier activity (GO: 0009055), peroxidase activity (GO: 0004601), defense response to fungus (GO: 0050832), voltage-gated anion channel activity (GO: 0008308), catalytic activity (GO: 0003824), response to salicylic acid (GO:0009751), etc. (Supplementary File S6).

We have concluded that the co-expression of *MSLs* with other genes involved in numerous biological processes suggested their diverse functions. The GO mapping of co-expressed transcripts suggested the participation of *MSL* genes in growth and development. For instance, one of *MSL* gene i.e. *HvMSL8* found to be co-expressed with ABC transporter, which was reported to be involved in pollen growth development^36^. Thus, *HvMSL8* can have probable role in pollen growth and development. We analyzed that *MSL* genes were co-expressed with cellulose synthase, suggested their involvement in cell wall development. Further, the co-expression of *MSL* genes with transcripts encoding kinases such as serine-threonine kinase, cysteine rich receptor kinase etc. suggested their roles in signaling pathways, and stress responses.

### Comparative expression profiling of paralogous genes

**Figure 5:**
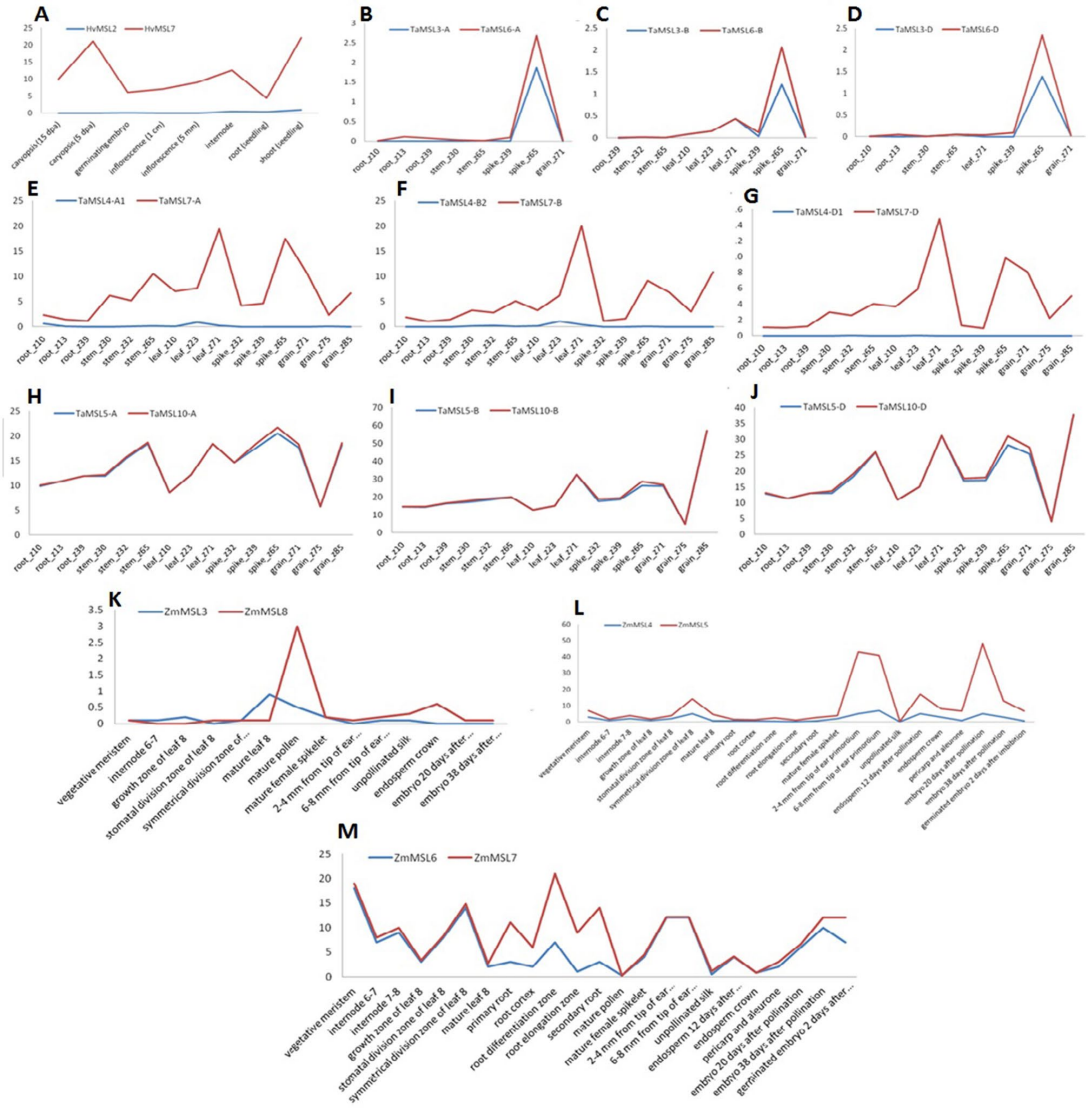
Comparative expression profiling of paralogous genes. (**A**,**B**–**J**,**K**–**M**) shows the comparative expression profiling of duplicated genes of *H. vulgaris, T. aestivum*, and *Z. mays,* respectively. Based on expression pattern, duplicated gene pairs have been classified into the retention of expression (**B**–**D**,**H**–**J**,**K**–**M**) and pseudo-functionalization (**A**,**E–G**).

The duplication events play a significant contribution to the origin of paralogous genes during evolution. Due to their participation in the formation of similar protein complexes, it is important to coordinate their expression within the cell^37^. Therefore, we performed comparative tissue expression profiling of paralogous *MSL* gene pairs using available high throughput RNA seq data^38–40^. The paralogous gene pairs could be divided into three classes; retention of expression, neo-functionalization, and pseudo-functionalization, based on their expression patterns. However, *MSL* paralogues genes exhibited only pseudo-functionalization and retention of functions, which suggested functional conservation after duplication. The segmentally duplicated gene pair of *H. vulgare* (*HvMSL2* and *HvMSL7*) exhibited pseudo-functionalization, due to the insignificant expression of one gene (Fig. 5A). However, in the case of *Z. mays*, three segmentally duplicated genes pairs such as *ZmMSL3: ZmMSL8; ZmMSL4: ZmMSL5* and *ZmMSL6: ZmMSL7* revealed their existence in class retention of expression due to their comparable expression pattern (Fig. 5K–M). Similarly, in *T. aestivum*, six segmentally duplicated gene pairs (*TaMSL3-A: TaMSL6-A; TaMSL3-B: TaMSL6-B; TaMSL3-D: TaMSL6-D; TaMSL5-A: TaMSL10-A; TaMSL5-B: TaMSL10-B; TaMSL5-D: TaMSL10-D)* also exhibited retention of function (Fig. 5B–D,H–J). However, three segmentally duplicated gene pairs *(TaMSL4-A1: TaMSL7-A; TaMSL4-B2: TaMSL7-B and TaMSL4-D1: TaMSL7-D)* showed pseudofunctionalization (Fig. 5E–G). Due to the non-availability of expression data, we could not perform the comparative expression analysis for paralogous genes of *Ae. tauschii* and *T. urartu*. Moreover, the results from above analysis revealed that despite of duplication during evolution, the function of paralogous *MSL* genes are still conserved.

### Expression profiling of *TaMSL* under biotic and abiotic stresses

Various environmental stresses such as abiotic and biotic stresses affect plant growth and development, which leads to the loss of yield and quality^41,42^. The expression patterns of *TaMSL* genes were analyzed to understand their role in biotic and abiotic stress responses.

**Figure 6:**
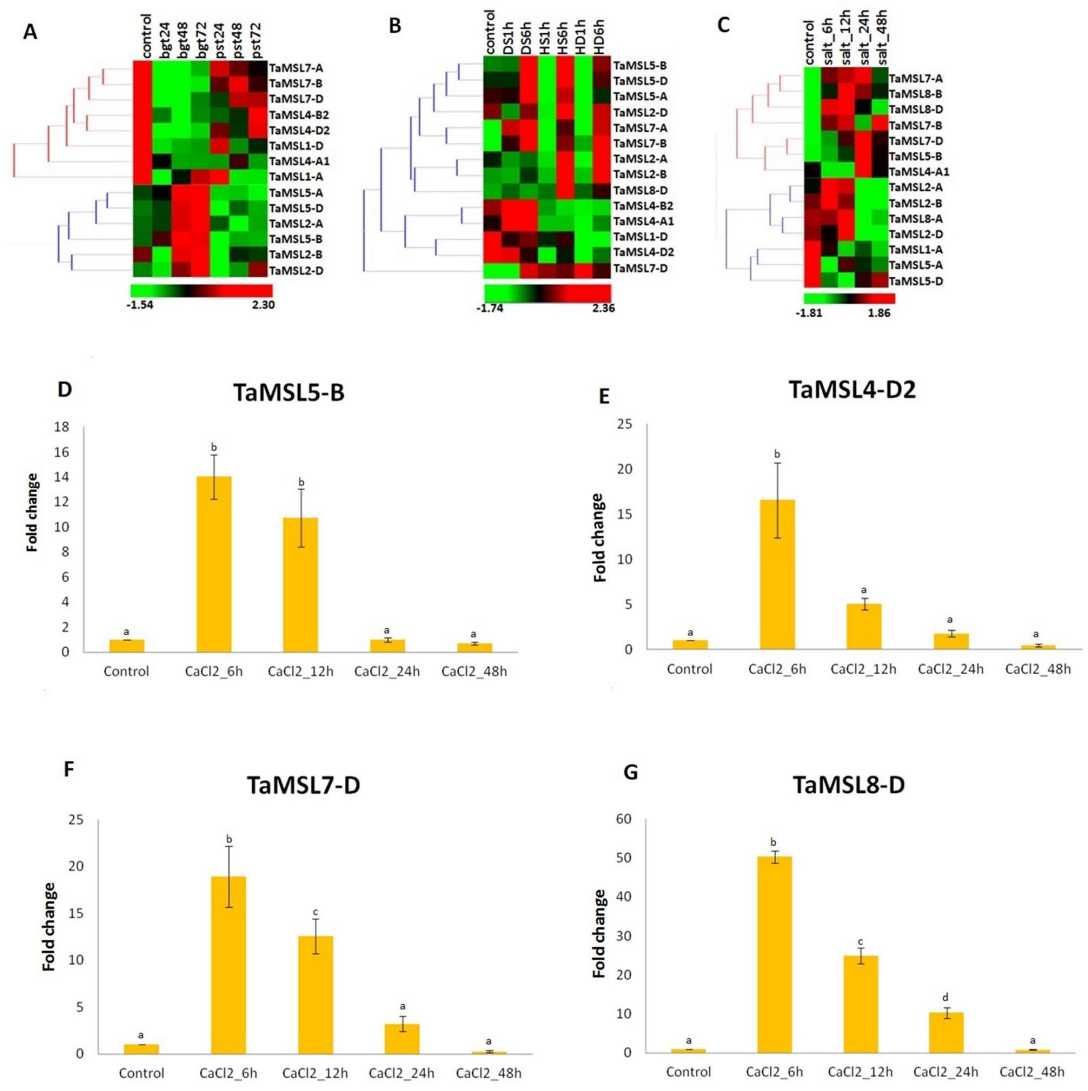
Expression profiling of *TaMSL* genes under biotic and abiotic stress conditions in *T. aestivum.* Heat maps (**A**–**C**) show the expression profile of up and downregulated genes under (**A**) biotic, (**B**) DS, HS, and HD and, (**C**) salt stress. Bar graphs (**D–G**) show the qRT-PCR results of four *TaMSL* genes under CaCl_2_ stress at 6, 12, 24, and 48 h of treatments. The bar graphs indicate the fold change of each *TaMSL* gene expression and a vertical line indicate standard deviation. A significant mean difference (≤ 0.05) level is shown by different letters on the top of each vertical bar.

Two fungal pathogens i.e. *Puccinia striiformis* f. sp. *tritici* (Pst) and *Blumeria graminis* f. sp. *tritici* (Bgt) were used for expression analysis of *MSL* genes under biotic stresses using high throughput RNA seq data^43^. The *MSL* genes, which showed a fold change of ≥ 2, were selected for the study (Supplementary File S7). *TaMSL* genes were clustered in two groups, based on their relatively similar expression. All *TaMSL* genes of group I was down regulated after Bgt and Pst infestation except *TaMSL1-A* which was up-regulated after 24 h of Pst attack. However, in group II, the majority of *TaMSL* genes were up-regulated after Bgt and down-regulated after Pst infestation. Moreover, *TaMSL2-B* and *TaMSL2-D* were down-regulated after 24 h of Bgt infestation and *TaMSL2-A*, *TaMSL5-D* and *TaMSL2-D *were up-regulated after 24, 48 and 72 h of Pst inoculation, respectively (Supplementary File S7, Fig. 6A).

We also analyzed the expression profiling of *TaMSL* genes under abiotic stress conditions such as drought stress (DS), heat stress (HS), heat drought (HD), and salt stress by using high throughput RNA seq data^44,45^. A total of 14 *TaMSL* genes were differentially expressed (≥ 2) under these stresses (Fig. 6B,C, Supplementary File S7). The majority of *TaMSL* genes were upregulated at later stages (6 h) of DS, HS, and HD treatment. However, a few genes such as *TaMSL7-A,* and *TaMSL7-B* were upregulated at 1 h of DS. *TaMSL7-D* and *TaMSL8-D* were upregulated at each hour of DS, HS, and HD treatment. In contrary, *TaMSL1-D* and *TaMSL4-D2* were downregulated in each treatment. Similarly, *OsMSL6* and *OsMSL3* of *O. sativa* show significant expression in response to drought stress^20^. Their orthologs, *TaMSL8-D, TaMSL5-A* and *TaMSL5-D* were also upregulated in drought stress (Fig. 6B).

Based on the similar expression patterns*, TaMSL* genes were clustered into two groups under salt stress (Fig. 6C). All *TaMSL* genes of group I showed upregulation during every hour of salt stress except *TaMSL4-A1,* which was downregulated at 6 and 12 h. In group II, the majority of *TaMSL* genes were downregulated, while certain genes such as *TaMSL2-A* and *TaMSL2-B* were upregulated at 6 h and 12 h of salt stress (Fig. 6C). *TaMSL5-B* was upregulated at every hour of salt stress treatment similar to its orthologs *OsMSL3* of *O. sativa*, which was also significantly upregulated during salt stress^20^.

The differential expression of *TaMSL* genes during various biotic and abiotic stresses along with the occurrence of various stress-responsive *cis*-regulatory elements including as-1, MYB, MYC, WRE3, GC-motif, DRE core etc., suggested their involvement in stress tolerance. All these stresses result in an increase in cytoplasmic Ca^2+^. Since the MSL proteins are also involved in Ca^2+^ transport, the differential expression of *TaMSL* genes along with their co-expression with various signaling related genes, suggested their roles in Ca^2+^ homeostasis and signaling during these stress conditions.

### Expression analysis during CaCl_2_ stress

Ca^2+^ is an important secondary messenger involved in signaling^46^. During stress conditions, the cytoplasmic Ca^2+^ concentration elevated by usually tenfolds, resulted in activation of signaling process and activation of various calcium transport elements to restore the cytoplasmic Ca^2+^ concentration by the process called calcium homeostasis^47^. Since the MSL proteins are also associated with the influx of Ca^2+^ and its homeostasis^16^, expression analysis of *TaMSL* genes was performed under CaCl_2_ stress using qRT-PCR. A total of four *TaMSL* genes (*TaMSL5-B* from class I, and *TaMSL4-D2, TaMSL7-D,* and *TaMSL8-D* from class II) were selected for qRT-PCR that represented each major phylogenetic group (Fig. 6D–G). The expression pattern of *TaMSL4-D2, TaMSL5-B, TaMSL7-D,* and *TaMSL8-D* was similar at every hour of CaCl_2_ stress. *TaMSL5-B*, *TaMSL7-D,* and *TaMSL8-D* were significantly upregulated (>  tenfolds) in the initial hours (6 and 12 h) of treatment. However, all these genes get normalized at later stages of treatment (Fig. 6D–G).

The elevated expression of class I *TaMSL* gene; *TaMSL5-B*, which was predicted to be localized in chloroplastic membrane, might be associated with the transport of Ca^2+^ from this organelle to the cytoplasm and stress signaling, as well. On the other hand, enhanced expression of class II *TaMSL* genes suggested that they might initially be activated and subsequently stimulated other Ca^2+^ channels through depolarization of the plasma membrane. These Ca^2+^ channels further help in Ca^2+^influx into the cytosol, which in turn activates various signaling pathways for the adaptation of plants under stress conditions. Further, the occurrence of various Ca^2+^ and phytohormone responsive *cis-*regulatory elements such ABRE, DRE, W-box etc. in the promoter region of *MSL* genes also suggested their involvement in Ca^2+^ homeostasis and signaling.

## Conclusions

The present study involved identification, classification and detailed characterization of MSL channels from six cereal crops. The occurrence of *cis-*regulatory elements in *MSLs* suggested their roles in the diverse physiological phenomenon. Expression profiling in various tissue developmental stages highlighted the role of *MSL*s in the growth and development. Co-expression analyses and gene ontology mapping revealed the plausible role of *MSLs* in signaling, stress responses, etc. Comparative expression profiling of paralogous genes showed either retention of expression or pseudo-functionalization. Abiotic and biotic stress responsive differential expression of *TaMSL* genes revealed their role in stress response. Enhanced expression of *MSLs* under CaCl_2_ stress suggested their involvement in Ca^2+^ homeostasis and signaling. However, the specific role of the individual *MSL* gene should be functionally validated in future studies.

## Methods

### Identification and nomenclature of *MSL* genes

To identify the MSL proteins in *Ae. taushii, H. vulgare, S. bicolor, T. aestivum, T. urartu* and *Z. mays*, a BLASTp search was performed at Ensembl Plants (plants.ensembl.org/index.html). The MSL protein sequences of *A. thaliana* and *O.sativa* were used as a query against the protein model sequences of these six cereals. Further, the MS-channel (PF00924) domain was confirmed in all the extracted MSL proteins by performing Pfam BLAST search (10^–10^) with Hidden Markov Model (HMM)^48^. This domain was further ensured within each MSL protein through the SMART^49^ and the NCBI Conserved Domain Database (CDD) BLAST servers^50^.

The international guidelines for gene symbolization were followed for the nomenclature of *MSL* of *T. aestivum* (https://wheat.pw.usda.gov/ggpages/wgc/98/Intro.htm). However, in case of *Ae. taushii, H. vulgare, S. bicolor, T. urartu* and *Z. mays* names were given to *MSL* genes according to their location on respective chromosome.

### Chromosomal localization, homeologs and paralogs analysis

Ensembl Plants was used to retrieve the information regarding the chromosomal and genome-wide distribution of *MSL* genes (https://plants.ensembl.org/Triticum_aestivum/). Chromosomal maps were prepared using Map Inspect (http://mapinspect.software.informer.com/), to depict the location of each gene on the respective chromosome. *TaMSL* genes of *T. aestivum* were grouped as homeologs based on their locations on the homeologous chromosomes and by performing bidirectional BLAST search with ≥ 90% sequence similarity. The paralogous genes formed at the time of duplication events were identified by using bidirectional blast search (10^−10^) with ≥ 80% sequence similarity. Based on the distance between the chromosomal localization of paralogous genes, they were considered as either segmentally or tandemly duplicated^51^.

### Ka/Ks calculations

The nucleotide and protein sequences of the duplicated *MSL* gene pairs were aligned using ClustalOmega server (https://www.ebi.ac.uk/Tools/msa/clustalo). Further, PAL2NAL server was used for the calculation of non-synonymous substitution per non-synonymous site (Ka), synonymous substitution per synonymous site (Ks), and Ka/Ks ratio^52^. The divergence time of each duplicated *MSL* gene pair was calculated by applying the formula T = Ks/2r, whereas T indicates the divergence time and r indicates the divergence rate. The value of divergence rate was implicated as 6.5 × 10^–9^ in cereals^53^.

### Multiple sequence alignment and phylogenetic relationship

Full-length amino acid (AA) sequences of each MSL protein were aligned via Multalin and MUSCLE programs^54^ to predict the conserved residues. The phylogenetic tree was generated by MEGA 7 software using the neighbor-joining method with 1000 bootstrap replicates^55^.

### Promoter analysis

An upstream promoter sequence (~ 1.5 kb) of each *MSL* was extracted from genomic sequences of all the studied cereal plants. By using the PlantCARE database, *cis*-regulatory elements were investigated among the extracted promoter sequences^56^.

### Gene structure analysis

The *MSL* gene structures were constructed by Gene structure display server (GSDS2.0) using genomic and coding sequences (CDS) of each cereal^57^. The structural features were visualized in form of intron/exon organization and presence of various intron phases.

### Protein characterization

Various physicochemical properties of MSL proteins such as MW and pI were calculated using standard parameters by the Expasy MW/pI tool^58^. Phyre2 tool was used to predict the number of TM regions in MSL proteins^59^. To determine the subcellular distribution of MSL proteins, the Prot comp 9.0 tool (https://linux1.softberry.com/berry.phtml) was used. Moreover, the Chlorop v1.1 program was used for the re-confirmation of chloroplastic localization of MSL proteins^60^. Multiple Expectation Maximization for Motif Elicitation (MEME- suite version 5.0.1), was used for the investigation of conserved motifs within MSL proteins^61^.

### Expression analysis of MSL genes

To study the expression patterns of *MSL* genes in various tissue development stages of *H. vulgare, S. bicolor* and *Z. mays,* the RNA seq data was retrieved from the expression ATLAS database^38^. However, in the case of *T. aestivum,* the publically available RNA seq data were obtained from NCBI and URGI databases^39,40^ (wheaturgi.versailles.inra.fr/files/RNASeqWheat/). Then, trinity package was used for the calculation of expression values in the form of FPKM (fragment per kilobase per million reads) in duplicates (n = 2)^62^.

The expression patterns were analyzed under biotic and abiotic stress conditions in *T. aestivum*. The expression of *TaMSL* genes was studied under biotic stresses by using publically available RNA seq data^43^ from leaves after 24, 48 and 72 h of fungal infestation of *Blumeria graminis f. *sp.* tritici* (Bgt) and *Puccinia striiformis f. *sp.* tritici* (Pst) in triplicates (n = 3). Expression study under abiotic stresses such as heat (HS), drought (DS), and their combination (HD) was done by using the available RNA seq data generated in duplicates (n = 2) after 1 and 6 h of these stresses from leaves^44^. Moreover, RNA seq data generated in triplicates (n = 3) by Zhang et al. in 2016, after 6, 12, 24, and 48 h of salt treatment from root tissues were used for expression profiling of *TaMSL* genes under salt stress^45^. Heat maps were prepared using Hierarchical Clustering Explorer 3.5^63^.

### Co-expression analysis and gene ontology mapping

The CoExpress v.1.5. tool was used for the analysis of co-expression of *MSL* genes of *H. vulgare, S. bicolor, T. aestivum* and *Z. mays*. The co-expression values were calculated by using the Pearson correlation coefficient^64^ with a correlation power 1 and threshold filter ≥ 0.9. Gene ontology (GO) mapping and functional annotation of co-expressed genes were done using the Blast2GO tool^65^. Interaction networks of co-expressed genes were made by the Gephi 0.9.1 tool^66^.

### qRT-PCR

Firstly, the surface sterilization of seeds of *T. aestivum* (cv. chinese spring) was done using sodium hypochlorite (1.2%) in 10% ethanol. Then, sterilized seeds were kept overnight at 4 °C for stratification after washing with double autoclaved water. The seeds were allowed to germinate at room temperature. After germination, these seedlings were placed in fresh phytaboxes and were grown in the plant growth chamber using autoclaved water. CaCl_2_ (20 mM) treatment was given to 7-day-old seedlings with growth media i.e. Murashige and Skoog (MS) and then, samples were collected in liquid nitrogen after 6, 12, 24, and 48 h. RNA was extracted using the Spectrum TM Plant Total RNA kit (Sigma, USA) from root tissues. Traces of DNA contamination were removed using the TURBO DNA-free™ Kit (Invitrogen, USA). RNA samples were examined both qualitatively and quantitatively through agarose gel electrophoresis and Nanodrop spectrophotometer, respectively. Superscript III First-Strand Synthesis Super-mix (Invitrogen, USA) was used for cDNA synthesis from one microgram of RNA. By using SYBR Green and gene-specific primers (Supplementary Table S1) of chosen *TaMSL* genes, qRT-PCR was done at CFX96 Real-Time PCR (BioRad, USA) by tracking already established protocol^67^. An ADP-ribosylation factor (*TaARF1*) was taken for the internal control and the delta-delta CT method (2-ΔΔCT) was used for calculating the expression values^68^. All the experiments were performed in triplicates (n = 3) and represented in term of mean ± standard deviation (SD). After Analysis of Variance, we performed the post hoc Tukey’s test at p-value < 0.05, to analyze the significant mean difference between control and treatments using SPSS 16.0 software.

## Supplementary information

Supplementary Information 1.

Supplementary Information 2.

Supplementary Information 3.

Supplementary Information 4.

Supplementary Information 5.

Supplementary Information 6.

Supplementary Information 7.

Supplementary Table S1.
