# Supplementary material for: Genome-wide characterization and expression analysis suggested diverse functions of the mechanosensitive channel of small conductance-like (MSL) genes in cereal crops
Source: Sci Rep. 2020 Oct 6;10:16583. doi: 10.1038/s41598-020-73627-7 (PMC7538590; doi:10.1038/s41598-020-73627-7)
Supplement: Supplementary file 8 — Supplementary Table S1. [file 41598_2020_73627_MOESM8_ESM.doc]

**Genome-wide characterization and expression analysis suggested diverse functions of the *mechanosensitive channel of small conductance-like* (*MSL*) genes in cereal crops**

**Amandeep Kaur^1^, Mehak Taneja^1^, Shivi Tyagi^1^, Alok Sharma^1^, Kashmir Singh^2^ and Santosh Kumar Upadhyay^1^***

**^1^**Department of Botany, Panjab University, Chandigarh, India-160014.

**^2^**Department of Biotechnology, Panjab University, Chandigarh, India-160014.

**Table S1.** List of qRT PCR primers.

| **Gene Name** | **5'-3'** |
| --- | --- |
| >TaARF_F | TGATAGGGAACGTGTTGTTGAGGC |
| >TaARF_R | AGCCAGTCAAGACCCTCGTACAAC |
| >TaMSL4-D2_F | CAACACAACCCAAGCAATTTTGCCGCG |
| >TaMSL4-D2_R | GTCGCCGTTCCCATCGATGTTCAGAA |
| >TaMSL5-B_F | GCATAACTCGTCCGGGAGGAACTACAT |
| >TaMSL5-B_R | AGGTACCACGAGTGCAGTAGCTGG |
| >TaMSL7-D_F | AAAATCCGGCCAGTCTCCTTCAGGG |
| >TaMSL7-D_R | AAGGGCTTTATCGTGAGGCTGCACACC |
| >TaMSL8-D_F | GATAAAGGAACGCCCAAGGTGATCGAT |
| >TaMSL8-D_R | ATTGGCTGCTGCCTTTGCCTCCC |
